# Sequential analysis of global gene expression profiles in immature and *in vitro *matured bovine oocytes: potential molecular markers of oocyte maturation

**DOI:** 10.1186/1471-2164-12-151

**Published:** 2011-03-16

**Authors:** Solomon Mamo, Fiona Carter, Patrick Lonergan, Cláudia LV Leal, Abdullah Al Naib, Paul McGettigan, Jai P Mehta, Alexander CO Evans, Trudee Fair

**Affiliations:** 1 School of Agriculture, Food Science and Veterinary Medicine, College of Life Science, University College Dublin, Dublin 4, Ireland; 2 Faculdade de Zootecnia e Engenharia de Alimentos, Universidade de São Paulo, Pirassununga-SP, Brazil

## Abstract

**Background:**

Without intensive selection, the majority of bovine oocytes submitted to *in vitro *embryo production (IVP) fail to develop to the blastocyst stage. This is attributed partly to their maturation status and competences. Using the Affymetrix GeneChip Bovine Genome Array, global mRNA expression analysis of immature (GV) and *in vitro *matured (IVM) bovine oocytes was carried out to characterize the transcriptome of bovine oocytes and then use a variety of approaches to determine whether the observed transcriptional changes during IVM was real or an artifact of the techniques used during analysis.

**Results:**

8489 transcripts were detected across the two oocyte groups, of which ~25.0% (2117 transcripts) were differentially expressed (p < 0.001); corresponding to 589 over-expressed and 1528 under-expressed transcripts in the IVM oocytes compared to their immature counterparts. Over expression of transcripts by IVM oocytes is particularly interesting, therefore, a variety of approaches were employed to determine whether the observed transcriptional changes during IVM were real or an artifact of the techniques used during analysis, including the analysis of transcript abundance in oocytes *in vitro *matured in the presence of α-amanitin. Subsets of the differentially expressed genes were also validated by quantitative real-time PCR (qPCR) and the gene expression data was classified according to gene ontology and pathway enrichment. Numerous cell cycle linked (*CDC2, CDK5, CDK8, HSPA2, MAPK14, TXNL4B*), molecular transport (*STX5, STX17, SEC22A, SEC22B*), and differentiation (*NACA*) related genes were found to be among the several over-expressed transcripts in GV oocytes compared to the matured counterparts, while *ANXA1, PLAU, STC1and LUM *were among the over-expressed genes after oocyte maturation.

**Conclusion:**

Using sequential experiments, we have shown and confirmed transcriptional changes during oocyte maturation. This dataset provides a unique reference resource for studies concerned with the molecular mechanisms controlling oocyte meiotic maturation in cattle, addresses the existing conflicting issue of transcription during meiotic maturation and contributes to the global goal of improving assisted reproductive technology.

## Background

Transition from the maternal to -embryonic genome control of development occurs relatively late in cattle, during the fourth cell cycle [1]. Thus, the oocyte is the main driver of early embryo development, drawing on maternal mRNAs and proteins accumulated during the oocyte growth phase [2,3]. In order to achieve a developmentally competent status, the oocyte has to gradually undergo a number of physiological changes that include physical and molecular remodeling [4]. During fetal life, mammalian oocytes initiate meiosis and become arrested at the diplotene stage of prophase I (dictyate stage). The ability of these oocytes to resume meiosis and to complete the first meiotic division is acquired sequentially during their growth phase [5]. In fully grown oocytes, meiotic resumption and nuclear maturation, in response to the preovulatory gonadotrophin surge *in vivo *or release from the follicle *in vitro*, is characterized by germinal vesicle breakdown (GVBD), chromosomal condensation, cumulus cell expansion, hyaluronic acid and cyclic AMP production [5-8], and progression through metaphase I to anaphase and telophase, with extrusion of the first polar body and arrest at metaphase II (MII) until reactivation at fertilization. The basic molecular machinery governing these developmental processes is relatively well conserved across mammalian species [9,10]; however, there is a clear difference in the timing of these processes between species (reviewed in [11,12]).

The origin of the oocyte and more specifically, the environment in which oocyte growth and maturation occur [13-17] has been implicated as an important determinant of the subsequent developmental competence of the oocyte. Transcriptional profiling of *in vivo *and *in vitro *matured (IVM) oocytes in cattle [18-20], humans [21], and rhesus monkeys [22] have shown variations in a number of genes and distinct pathways, which may have consequential effects during the post fertilization development. For example during the process of *in vitro *embryo development, while maturation and fertilization proceed apparently normally (based on first polar body extrusion and mitotic cleavage, respectively), the proportion of embryos reaching the transferable (blastocyst) stage rarely exceeds 40 to 50% and those that do reach this stage are often compromised in quality and further developmental competence [23], an effect partly attributed to inadequate oocyte cytoplasmic maturation [24,25]. In contrast, fertilization and culture *in vitro *of oocytes matured *in vivo *results in high rates of blastocyst development [15,26] providing further evidence of the importance of oocyte quality in determining developmental competence.

To date, efforts to characterize developmentally competent oocytes have been hampered partly by the difficulty in assessing cytoplasmic maturation [27], and the small volume of material available for analysis. Currently, while nuclear maturation can be assessed by the extrusion of the first polar body and the formation of the second metaphase plate, there is no reliable assay for the assessment of oocyte cytoplasmic maturation, other than the development of the fertilized oocyte to a live offspring [24,27,28]. This lack of oocyte quality markers has led some researchers to use embryo morphology or blastocyst rate as alternative early quality predictors of developmental competence [29-31]. Until the mechanisms involved in establishing oocyte quality are elucidated, any effort to use assisted reproductive technologies in the treatment of human infertility or in animal production will be inefficient [28]. Analysis of the oocyte transcriptome during maturation using global mRNA analysis techniques provides a resource that can be mined and expanded continuously with new software and sequencing techniques to identify genes involved in the processes of meiotic and cytoplasmic oocyte maturation and the specific checkpoints regulating acquisition of full competence [32].

It is well understood that mRNAs stored during the oocyte growth phase are systematically, and in a step-wise manner, degraded or translated to form proteins that regulate subsequent developmental processes [33,34]. As a result, down regulation of stored transcripts during oocyte maturation is believed to occur. Although GV intact oocytes show some transcriptional activity [4,35], there are conflicting reports on the occurrence of transcription following the resumption of oocyte meiotic maturation. Comparison of immature (germinal vesicle stage, GV) and *in vivo *matured oocytes in mice [36], and in humans [32] indicated up regulation of a number of transcripts during oocyte maturation. However, other studies comparing immature and *in vitro *matured (IVM) oocytes in cattle [37] and in mice [38] reported the absence of over-expressed transcripts during oocyte maturation. Given these apparently conflicting observations, and the opportunity to benefit from the increased coverage (more than 92%) of the bovine genome sequence [39], we examined transcriptional activities during bovine oocyte maturation and present a dataset that provides a unique reference resource for studies concerned with the molecular mechanisms controlling oocyte meiotic maturation in cattle. These data have been superficially referred to in a previous review paper [2] but a thorough analysis of the gene lists and associated ontologies has not yet been published.

## Results

### Experiment 1 Global oocyte transcriptome analysis revealed differentially regulated transcripts

The hybridized slides were scanned and MIAME-compliant (FGED; http://www.mged.org) gene expression data have been submitted to the Gene Expression Omnibus (GEO) database (GSE23449).

The established Affymetrix linear amplification procedures using the GeneChip^® ^Expression 3'-Amplification Two-Cycle cDNA Synthesis kit yielded closely similar profiles between the five replicates of the same treatment (Pearson's correlation coefficients of the range 0.914 to 0.949), indicating a highly reproducible procedure. Moreover, hierarchical clustering and principal component analysis revealed sufficient differences between the transcriptomes of immature and IVM oocytes that allowed them to cluster separately into two groups based on maturation status with a high degree of reproducibility and small variability between samples of each treatment (Additional file 1, Figure S1 and Additional file 2, Figures S2A and S2B). The first principal component (PCA1) accounts for 95.12% of the variability.

**Figure 1:**
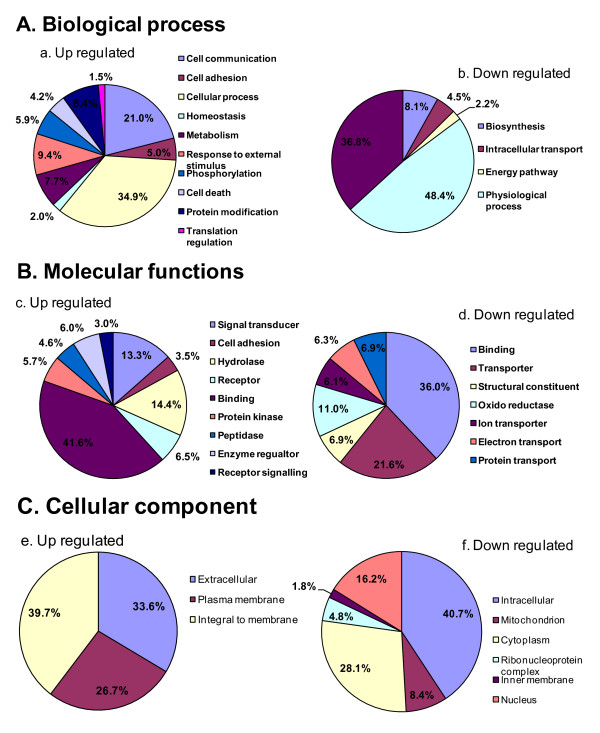
**Classification of the differentially expressed transcripts based on gene ontology**. (A) Biological process (a and b); (B) Molecular functions (c and d); and (C) Cellular components (e and f)

By comparing multiple probes matching a target mRNA and evaluating the signal-to-noise ratio, the FARMS http://www-stat.stanford.edu/~tibs/SAM/sam.pdf microarray processing algorithm detected a total of 9178 informative *Bos taurus *probes corresponding to 8489 annotated genes. 2117 transcripts were found to be differentially expressed between immature and IVM oocytes, corresponding to 1528 transcripts that were significantly lower and 589 that were significantly higher in abundance in IVM oocytes compared to their immature counterparts. The list of differentially expressed transcripts was further analyzed using the DAVID Bioinformatic Resource http://david.abcc.ncifcrf.gov/home.jsp and was found to correspond to 1836 annotated transcripts with NCBI Entrez-Gene IDs, of which 1413 were under-expressed and 423 were over-expressed in the IVM oocytes. The summary and details of differentially expressed transcripts are described in Table 1, Additional file 3, Table S1 and Additional file 4, Table S2. Transcripts were classified according to their gene ontology (GO): molecular function, cellular component and biological process, and the results of overrepresentation analysis are presented in Figure 1.

**Table 1:** Summary of the global oocyte transcriptome analysis showing the differentially regulated transcripts during bovine oocyte maturation

Category	Probe sets	Transcripts	Unique annotated Transcripts
Oocyte Transcriptome	9178	8489	6586
Differentailly expressed	2244	2117	1836
Increased in MII	613	589	423
Increased in GV	1631	1528	1413

Biological processes enriched with differentially regulated genes included regulation of various cellular processes, cell communication and intracellular transport, metabolism and translational regulation (Figure 1a and 1b). The majority of over expressed transcripts were associated with cell communication and various cellular processes including homeostasis (Figure 1a) while the metabolic and intracellular transport processes (Figure 1b) were primarily enriched by transcripts which were under expressed in IVM oocytes. This finding is supported by the results of an earlier study in mouse which showed that chemical inhibition of some metabolic pathways induced oocyte maturation [40]. Pathway analysis of the differentially expressed genes using the Ingenuity Pathway Analysis tool detected five major networks, preferentially populated with genes that have important biological functions relevant to development, including cellular growth and development, Molecular transport, Protein synthesis and embryo development (Table 2). Moreover, the detected networks were supportive of the above listed GO results.

**Table 2:** Top network functions detected during Ingenuity Pathway Analysis

Associated Network Functions	Score
1. Cellular assembly and organization, Molecular transport, Protein Trafficking	42
2. Protein synthesis, Cellular function and Maintenance, Small molecule Biochemistry	35
3. Post-Translational Modification, Developmental Disorder, Embryonic Development	35
4. Genetic disorder, neurological disease, Cell-to-Cell Signalling and interaction	35
5. Cancer, Neurological disease, Renal and Urological Disease	35

### Experiment 2 Quantitative PCR (qPCR) validation of microarray results

**Figure 2:**
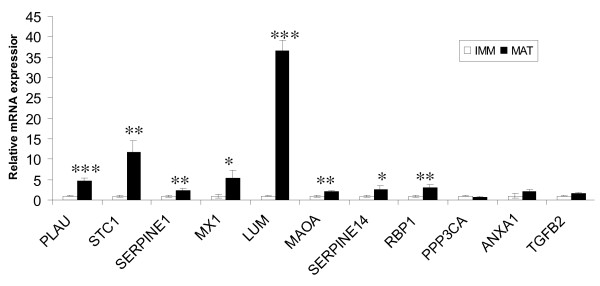
**qPCR analysis of selected genes from the list of over-expressed transcripts**. Expression of MII oocytes (black bars) was compared to GV oocytes (white bars). All expression levels are relative to the level of expression in GV oocytes (white bars) which has been arbitrarily set to one-fold. Stars denote statistical difference * = p < 0.05, **= p < 0.01, ***= p < 0.001.

**Figure 3:**
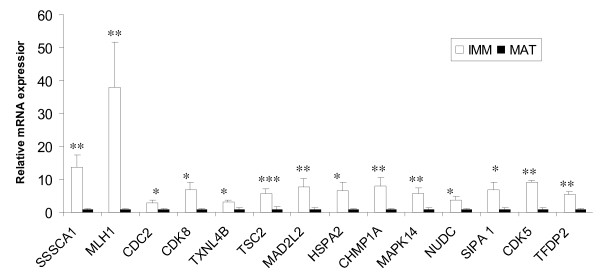
**qPCR analysis of selected genes from the list of under-expressed transcripts**. Expression of MII oocytes (black bars) was compared to GV oocytes (white bars). All expression levels are relative to the level of expression in MII oocytes (black bars) which has been arbitrarily set to one-fold. Stars denote statistical difference * = p < 0.05, **= p < 0.01, *** = p < 0.001.

A panel of 25 differentially expressed genes mostly associated with cell cycle functions, and a reference gene (*H2AFZ*) (Table 3) was selected for validation in independently prepared immature and IVM oocyte samples using qPCR. All but one of the genes (24/25) showed a similar expression pattern to the microarray data and 22 of the 25 genes were significantly differentially expressed (P < 0.05) (Figures 2 and 3).

**Table 3:** Sequences and GenBank accession numbers of primers used for qPCR analysis during experiment 2 and 3.

Gene	Forward 5'-3'	Reverse 5'-3'	Product size (bp)	GenBank
*ANXA1*	GAGGAAGTTGTTTTGGCTCTATTGA	TGGCAGCACGGAGCTCTT	67	NM_175784
*CDK1*	TGGACAGTCAAATTAAGAAGATGTAGCT	GTACAATTATCTGCTCTTGACACAACAC	72	NM_174016
*CDK5*	CCTGCTCATCAACAGGAATGG	AAA GGC GCG AGC CAAAC	61	NM_174017
*CDK8*	CCCAGCAGCCTCCACAGTA	TGT CCG ACG CAG CTC AGT AC	57	XM_583707
*CHMP1A*	CAGCCCTGGGACTCTTCTTCT	ACTCACCCTGTAGGGCACAGA	62	NM_001037584
*H2AFZ*	TCCGGAAAGGCCAAGACA	GAACTGCAAACCGGCTCTCT	57	NM_174809
*HSPA2*	AGAACCAGGTGGCCATGAAC	TCCGACCAATCAGCCTCTTG	63	NM_174344
*LUM*	TTCAAAGCATTCGCCAAAATG	CCGCCAATTAATGCCAAGAG	62	NM_173934
*MAD2L2*	GTCTACCCGGTGGGCATCT	CAT CTG GAC AGG CAC GTT GT	57	NM_001045946
*MAOA*	GGGCCAGATGTTCGACGTAGT	AGCTAAGAGTTTCGCAGCAGATAA	69	NM_181014
*MAPK14*	GCT GTC GAC CTG CTG GAG AAG ATG	TCG TCG TCA GGA TCG TGG TAC TGG	110	NM_001102
*MLH1*	TCCGGGAGATGCTGCATAA	CAA GGC CCA CTG AGG ATT CA	59	NM_001075994
*MX1*	GAGCAGTATGACTCCCGACTGTTT	TTCTCAACCACAGCACTCCATTT	71	NM_173940
*NUDC*	CCCCAGATCAAAGAACTGACTGA	GGC ATC CTT TTT CTG GTC AATT	72	NM_001075607
*PLAU*	CGCCACACACTGCTTCATTG	GCCGTGACTGACCCAAGTAGAC	68	NM_174147
*PPP3CA*	CCTCATCCATACTGGCTTCC	AATCCATCTTCTTCTGACCC	140	NM_174787
*RBP1*	TGCGCGCGCTGGAT	GCTTCAGCAAGTTGGCGATT	54	NM_001025343
*SERPINE1*	GGACTTCTCCAGTTTTTCAGATCAAG	CAGCGTGCCGCTCTCATT	91	NM_174137
*SERPINA14*	ACCCTGAGGACCTGAAACTTGAG	CGTGGACCAGAGGCTGTAAGTACT	69	NM_174797
*SIPA1*	GCCCATTATTCGGTGACACAGT	GAAGTCCCCTTTTCTCCACAGA	72	NM_001101895
*SSSCA1*	TGACTGCGGGACGATCCT	TGA CAA GCC ACG CAG TAG ATT T	60	NM_001038528
*STC1*	TTTGCAATGGCGGCATT	TCCCGAGGAGAGGCATAGAG	60	NM_176669
*TFDP2*	GGTGCCACCTTGTCCCAAT	CTA AGG CCA CTT CAG CAT CCA	64	NM_001075241
*TGFB2*	TCTCCAACCCAGCGCTACA	TTCACCCTCTGCTCTGGTTTTC	57	NM_001113252
*TSC2*	CAGAGGGCAAACAGACTGAGTTTAT	GCG ATT ATT GAG GCC ACA TTC	80	XM_581197
*TXNL4B*	TGGTAATCGTGATGCATGGAA	GGC TGG TTG CCT CAT GGT	58	NM_001014897

### Experiment 3 Effect of α-amanitin on transcript abundances

**Figure 4:**
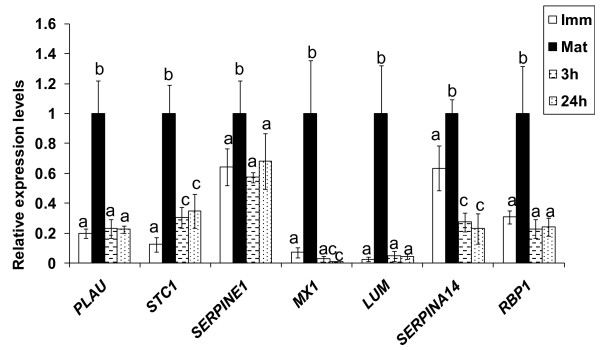
**Relative abundance of specific transcripts in bovine oocyte matured with or without α-amanitin**. The figure shows GV (white), MII (black), MII oocytes matured in the presence of α-amanitin after 3hr of culture (line spotted), MII oocytes matured in α-amanitin for 24 hr (dot spotted). All expression levels are relative to the level of expression in MII oocytes (black bars) which has been arbitrarily set to one-fold

α-amanitin is known as a transcription inhibitor and used in various oocyte and embryo development studies [41,42]. Following incubation with the transcription inhibitor, α-amanitin, the expression of selected genes over-expressed in the microarray analysis (*LUM, MX1, SERPINA14, STC1, PLAU, SERPINE1*, and *RBP1*) was analyzed with qPCR, and transcript abundances were compared. Treatment with α-amanitin for either 21 or 24 h significantly reduced transcript abundances for all genes studied, compared to oocytes matured for 24 h in the absence of the inhibitor (Figure 4). Furthermore, for many of the transcripts analyzed, the expression profile of the α-amanitin-treated oocytes was similar to that of the immature oocytes.

### Experiment 4 Effects of analysis parameters on the transcript abundances

#### 4a Contribution of RNA conversion methods to differential expression data

The aim of this experiment was to examine whether the primers used during cDNA synthesis have contributed to the conflicting data on transcription during mammalian oocyte maturation. Primers used during the reverse transcription reaction are known to influence the cDNA quality and yield. Unlike random primers, oligo (dT) primers tend to show a bias towards the 3' end of transcripts with poly (A) tails. In order to investigate the effect of the primer used during cDNA synthesis on qPCR analysis data, we compared the transcript abundances of a panel of genes in two cDNA preparations obtained from reverse transcription using either random or oligo (dT)-based primers. The transcripts were quantified and normalized to the quantity of an exogenous reference (luciferase), and reported as fold change differences relative to the immature oocyte transcript abundance.

Generally for an equal quantity of cDNA input, transcripts of the same gene were more abundant in random-primed cDNA preparations compared to oligo (dT)-primed preparations, as manifested by an earlier Cq value for the former compared to the latter. The expression patterns were similar for both random- or oligo (dT)-based priming. However, the calculated ratios for the oligo (dT)- primed cDNA showed a tendency for higher fold change differences for most genes compared to the random hexamer primed cDNA, but differences were not significant (Additional file 5, Figure S3). Moreover, there was no significant difference in the results generated by the use of primers for longer amplicons (Table 4) compared to those for shorter amplicons (Table 3), indicating no bias due to product size in the current study.

**Table 4:** Sequences and GenBank accession numbers of re-designed primers used for qPCR analysis during experiment 4.

Gene	Forward 5'-3'	Reverse 5'-3'	Product size (bp)	GenBank
*ANXA1*	ATGGTATCTGAATTCCTCAAGCAG	TGCAAGGCCTCAACATCC	149	NM_175784
*H2AFZ*	CGGAAAGGCCAAGACAAAG	CTGAAATCTAGGACGACTAGCCAT	103	NM_174809
*Luciferase*	TCCTCCAGGGATACGACAAG	GGTATCCAGATCCACAACCTTC	139	EU684088
*LUM*	CGAAAGCAGGGTCAAGACAG	TGATGACCTCCCATACAGTGC	158	NM_173934
*MAOA*	TGGCGGACCATGGATAAC	AAACTGCCGAGCAGTCTTTG	138	NM_181014
*PLAT*	TGTGGAGCTGTCTTCACGTC	CGTGTTGGCGGTACGTC	118	NM_174146
*PLAU*	CTGTGCCCTGGTCGTGAG	GCAACTGCATCGCTGAATG	136	XM_174147
*PPIA*	CCACCGTGTTCTTCGACATC	CCAAATCCTTTCTCTCCAGTGC	130	NM_178320
*SDHA*	GGGAGGACTTCAAGGAGAGG	TCAACGTAGGAGAGCGTGTG	112	NM_174178
*SERPINE1*	CAGGCGGACTTCTCCAGTT	CATTCGGGCTGAGACTACAAG	135	NM_174137
*STC1*	GTGACACAGATGGGATGTACGAC	CGAATGGCCAGGAAGACC	142	NM_176669
*TGFB2*	AGGCCGAGTTCAGAGTCTTTC	TGTAGCGCTGGGTTGGAG	117	NM_001113252
*YWHAZ*	GCAGATGGCTCGAGAATACAG	GAAGCGTTGGGGATCAAG	102	NM_174814

#### 4b Chronological analysis of candidate gene abundance during in vitro oocyte maturation

To avoid the possibility of preferential amplification due to poly (A) tail length, we standardized the cDNA synthesis procedures by using random primers that can account for transcript abundances, irrespective of the poly (A) tail status.

**Figure 5:**
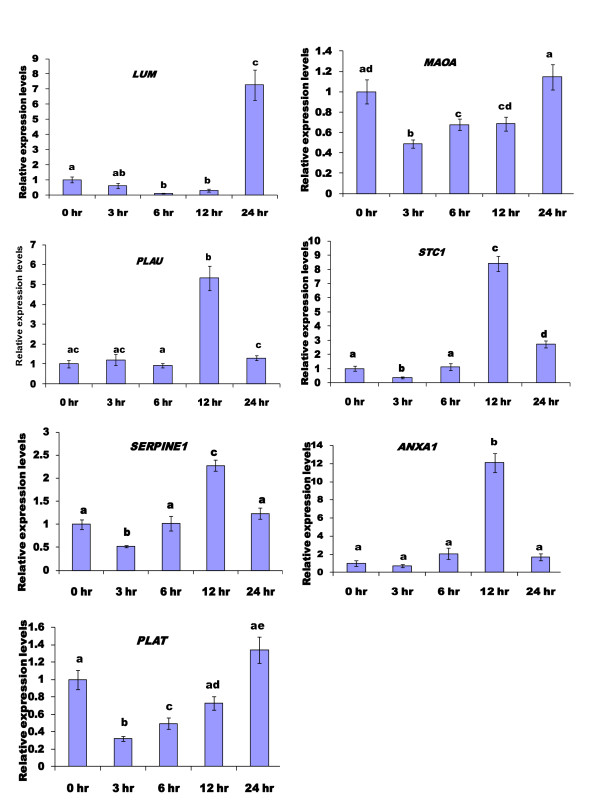
**Relative gene expression profiles of *in vitro *mature bovine oocytes at different time points**. In all cases the expression at time 0 h was taken as calibrator against which the relative levels of other time points were calculated. Time points with the same letter are not significantly different (p < 0.05).

In order to characterize in more detail the chronology of the divergence in transcript abundance between immature and IVM oocytes, oocytes were collected at 0 (GV), 3, 6, 12 and 24 (MII) h after initiation of IVM. The expression profiles of the over-expressed transcripts across the 24 h maturation window are presented in Figure 5. The relative abundance of four genes (*MAOA, STC1, SERPINE1 *and *PLAT*) was significantly (p < 0.05) reduced after 3 h maturation and gradually increased thereafter to peak either at 12 h (*STC1 *and *SERPINE1*) or 24 h (*PLAT *and *MAOA*). The expression levels of (*ANXA1, PLAU *and *LUM*) did not change after 3 h maturation, but increased thereafter to peak significantly (p < 0.05) at 12 h (*ANXA1 *and *PLAU*) or 24 h (*LUM*). The results also indicated that expression levels of *SERPINE1, STC1, PLAU, and ANXA1*) were peaked at 12 h post initiation of IVM and values were significantly (p < 0.05) higher than those observed at all other time points. Although abundance of these transcripts tended to increase in 24 h IVM oocytes, compared to 0 h, only expression of *LUM *and *STC1 *were significantly higher (p < 0.05). In general, the expression levels observed at 24 h were similar (but not identical) with the earlier profile for the same gene at this time point following normalization using luciferase. This similarity reinforced our approach to use validated reference genes for this and other subsequent experiments.

#### 4c Candidate gene expression in oocytes matured in vivo

The aim of this experiment was to verify if the gene expression changes observed in the *IVM *oocytes (Experiment 4b above) are also observed in oocytes matured *in vivo*.

**Figure 6:**
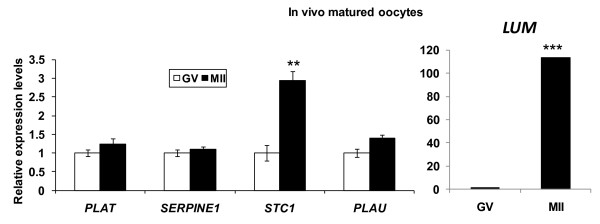
**Relative gene expression profiles of bovine oocytes, before (GV) and after (MII) *in vivo *maturation**. In all cases the expression at time immature (GV) stage was taken as calibrator against which the relative levels of other time points were calculated. ** indicates significance (p < 0.01) and *** indicates significance (p < 0.0001). Due to the insignificant levels of *LUM *in immature oocytes an arbitrary 40 Cq values were assigned to calculate the relative fold change.

The expression profiles of five transcripts (*LUM, PLAT, SERPINE1, STC1 *and *PLAU*) that were over-expressed in oocytes matured *in vitro *were quantified in *in vivo *derived bovine oocytes. The results revealed a similar tendency of increased abundance after maturation for all examined genes (*PLAT, SERPINE1, STC1 *and *PLAU*); the differences were significant in the case of *STC1 *(*p *< 0.01) and *LUM *(*p *< 0.00001) (Figure 6). The significant over-expression of these genes (*STC1 *and *LUM*) in mature oocytes was similarly noted in *in vitro *samples (Figure 5). Due to the negligible expression of *LUM *in immature oocytes an arbitrary Cq value of 40 was assigned for the expression in immature oocytes, in order to calculate the relative abundances.

## Discussion

The issue of transcript regulation during oocyte maturation is a controversial topic in developmental biology. Various previous efforts [32,36-38] to decipher the transcriptional changes during oocyte maturation have been masked by contradictory outcomes. In the current study, we established the global changes in transcriptomic profile during meiotic maturation in bovine oocytes. Moreover, in an effort to examine the possible sources of conflicting reports, we further investigated transcription in the presence of transcription inhibitor, effects of primers, amplified product sizes, reference genes and sample types (*in vitro *and *in vitro*) on transcript abundance. Here we (1) report the existence of transcription activities during bovine oocyte maturation, (2) show suppression of over-expressed genes when oocytes were matured in the presence of an inhibitor of transcription suggesting that the observed transcript changes were newly synthesized, (3) report that the new transcription was not sample type (*in vitro *or *in vivo*) -specific, (4) report primers used during cDNA synthesis and reference genes used for normalization have an impact on the interpretation of the gene expression data, but not the amplified product sizes.

The genome-wide interrogation of immature and *in vitro *matured bovine oocytes on the Affymetrix GeneChip bovine array identified a number of differentially expressed genes, the vast majority (~75%) of which were over-expressed in immature oocytes (Table 1). It is well accepted that mammalian oocytes have already accumulated the majority of their transcripts at the fully grown immature (GV) stage that will drive subsequent development through degradation, translation and post-transcriptional modifications [33,34] up to embryonic genome activation. Therefore, the observation of a massive reduction of the initial transcript stock in our study was in line with the current understanding of events during oocyte maturation. On the other hand, the detection and confirmation of certain over-expressed transcripts during bovine oocyte maturation suggests the existence of transcription, perhaps to complement the depleting transcriptional stock.

Despite the multiple roles of a particular gene [43], Ingenuity Pathway Analysis classified the differentially regulated genes into various associated functional network groups (Table 2). These include cellular assembly, molecular transport, post-translational modification and cell to cell signaling, all of which occur during oocyte maturation. For example, cell-to-cell signaling between oocytes and their surrounding somatic cells is important for oocyte cytoplasmic maturation and the acquisition of developmental competence. This is a bidirectional communication mediated through the transport of various growth factors, such as *GDF9 *and *BMP15*, from oocytes to their surrounding cumulus cells [44-46], and cyclic adenosine monophosphate (cAMP) from somatic cells to the oocyte [47] via gap junctions.

Generally, the sequence of events leading to the GVBD (Germinal Vesicle Breakdown) and the requirements for transcription and/or protein synthesis differs markedly between species [12,48]. For example, in frog, mouse, rat and fish oocytes, high levels of cAMP prevent oocyte maturation *in vitro*, while a decrease in oocyte cAMP is associated with the resumption of meiosis [49,50]. In contrast, maturing oocytes from pig, sheep, cattle and rabbit exhibit a transient increase rather than a decrease in cAMP levels, and treatments that increase cAMP levels can induce oocyte maturation [49,51-53]. Similarly, an earlier study [12] confirmed the requirements of transcription and protein synthesis as requirements for GVBD in domestic animals (sheep, cattle and pigs) while neither event is required for the initiation of maturation in mouse oocytes. Generally our finding is in line with earlier studies in bovine [54-56] that observed various over-expressed transcripts during oocyte maturation.

In order to verify if the over-expressed transcripts were transcribed following submission of oocytes to IVM, oocytes were matured in the presence of the RNA polymerase II inhibitor, α-amanitin for 24 h. The resulting transcript profile was similar to that of the immature oocytes, which is consistent with the notion that 24 h exposure to α-amanitin prevents meiotic resumption in most oocytes [57]. This study [57] also reported that addition of α-amanitin after 3 h of culture had no effect on meiotic maturation. However, in the current study exposure of the oocytes to the α-amanitin treatment following an initial 3 h culture in α-amanitin-free medium resulted in a similar level of expression to that observed when the inhibitor was present throughout. These findings suggest that some de novo transcription is occurring in bovine oocytes following the resumption of meiosis.

In order to examine the contributions of some downstream analysis procedures on the final transcript data, we further examined the implications of primer choices during cDNA synthesis. Although not significant, there was a tendency for higher expression fold change (ratio) for oligo (dT)-based cDNA preparations compared to random-based cDNAs. This suggests the preferential amplification of oligo (dT)-based primers, and the finding is in agreement with most other previous observations [54,58-61], although another study [19] reported an identical results irrespective of the primers used. The fact that anchored oligo (dT) was used in our study may have narrowed the difference. It has been shown previously that anchored oligo (dT) primers are better than the conventional oligo (dT) primers in maintaining the fidelity of the probes, as the latter generates a high frequency of truncated cDNA through internal poly (A) priming [62]. This observation further signifies the contribution of primers to the final conclusions, and the need to select appropriate primers commensurate with the sample type for analysis. Therefore, it is possible to speculate that this may have also contributed to the earlier contradictory reports on the occurrence of transcription during meiotic maturation [32,36-38].

Increasing stringency by controlling primers for cDNA synthesis, designing intron-spanning primers for amplification and the use of alternative validated reference genes during the analysis appears to reduce the number of significant genes at 24 h. Interestingly, when we analysed the kinetics of transcript expression during maturation the abundance of several transcripts was significantly higher at 12 h compared to 0 h. The kinetics of bovine oocyte maturation has been well described [63-66]. In most oocytes GVBD occurs between 4 and 8 h after initiation of maturation, and has occurred in the majority of oocytes by 8 h. By 12 h the majority of oocytes have reached metaphase I. Progression from GVBD through the subsequent stages of meiosis is under the control of the anaphase promoting complex (APC) which is mainly regulated through sequential polyadenylation and deadenylation of transcripts [67] and the increased abundance of these transcripts at this time may reflect their association with APC. These genes are implicated in various developmental activities including cell signaling, apoptosis and membrane trafficking (*ANXA1*) [68], and cumulus cell expansion (*PLAU*) (reviewed in [55]).

Irrespective of the introduction of increased stringency measures, the abundance of *STC1 *and *LUM *was significantly higher in both *in vivo *and *in vitro *matured oocytes compared to 0 h (GV stage oocytes) (Figure 5 and Figure 6). Furthermore, transcript abundances were maintained at 0 h levels when oocytes were matured in the presence of α-amanitin. Taken together, these findings strongly suggest de novo transcription of *STC1 *and *LUM *following the resumption of meiosis. Analysis of various studies suggests *STC1 *has effects on metabolism, reproduction, and developmental processes in addition to affecting mineral homeostasis (reviewed in [69]. *STC1 *expression was highest in mouse ovary, with lower but detectable levels in most other tissues [70]. Based on this result and the initial implication of the gene in mineral metabolism, it was suggested that *STC1 *may have acquired an important function in reproduction during its evolution in mammals [70]. Similarly, increasing evidence suggests that *LUM *may also serve as a regulatory molecule of several cellular functions [71,72]. Previous study in mice using Northern and In situ hybridization indicated that, in early stages of embryonic development before day 7 post-coitus, the embryo does not express *LUM *or expresses only very low amounts [73]. This is the first study to examine and reveal the expression of these two genes (*LUM *and *STC1*) during oocyte maturation. Based on the consistent expression pattern in repeated experiments of *in vitro *and *in vivo *derived oocytes, it is plausible to speculate that these two genes (*LUM *and *STC1*) may be potential molecular markers of oocyte maturation and may contribute to the early events of embryo development.

## Conclusions

We have used global microarray analysis to establish the molecular transcriptome blueprint of immature and matured oocytes and to identify and validate genes that are unique to and predominantly expressed in bovine immature and IVM oocytes. The genes identified will be invaluable in further studies examining the processes of oocyte maturation in addition to addressing the existing conflicting issue of transcription during meiotic maturation. Moreover, it will enable the comparisons across many species and contributes to the goal of improving assisted reproductive technology.

## Methods

All chemicals, unless stated otherwise, were purchased from Sigma-Aldrich Chemical Inc. (St. Louis, MO, USA). All experimental procedures involving animals were licensed by the Department of Health and Children, Ireland, in accordance with the Cruelty to Animals Act (Ireland 1897) and the European Community Directive 86/609/EC and were sanctioned by the Animal Research Ethics Committee of University College Dublin. Four experiments each with different objectives and methodologies were carried out, as described separately in the result sections.

### Collection of immature and *in vitro *matured oocytes

Immature cumulus oocyte complexes (COCs) were obtained by aspirating 3 to 8 mm follicles on the ovaries collected from cows slaughtered at a local abattoir. Good quality COCs, judged morphologically with multiple cumulus layers and homogenous cytoplasm, were selected under the stereo microscope and washed repeatedly in modified phosphate-buffered saline (PBS, supplemented with 36 mg mL^-1 ^pyruvate, 50 mg mL^-1 ^gentamycin, and 0.5 mg mL^-1 ^bovine serum albumin fraction V). Following washing, half of the COCs were immediately denuded by repeated pippeting in PBS. After carefully evaluating the oocytes under a stereomicroscope, intact oocytes with no cumulus traces were pooled in groups of 10 oocytes per tube and immediately snap frozen in liquid nitrogen. The remaining half COCs were incubated in maturation medium [TCM-199 supplemented with 10% (v/v) fetal calf serum and 10 ng/ml epidermal growth factor] in 4-well dishes (Nunc, Roskilde, Denmark) at 39°C for 24 h under an atmosphere of 5% CO_2 _in air with maximum humidity. At 24 h, *in vitro *matured (IVM) COCs were denuded and snap frozen as described above. Five replicates (= days of ovary collection) were carried out, and all samples were stored at -80°C until analysis.

### Collection of immature and *in vivo *matured oocytes

The collection of immature and *in vivo *matured oocytes was carried out as previously described [26,74,75]. Briefly, ten crossbred beef heifers were synchronized using an 8-day CIDR treatment with administration of a prostaglandin F2a analogue (PG), the day before CIDR removal, to ensure complete regression of the corpus luteum. Animals were checked for standing estrus (= Day 0) and starting on Day 10 of the estrous cycle, animals were superovulated with FSH given as twice daily injections over four days. Luteolysis was induced with a second PG injection given on Day 12. To collect immature COCs, five cows were slaughtered 40 h after PG, equivalent to the expected time of the LH surge, and therefore just before resumption of meiosis. The remaining five cows received GnRH at 40 h post PG to induce an LH surge [15,76], and were slaughtered 20 h later (i.e. 60 h after PG) to collect *in vivo *matured COCs. 32 good quality *in vivo *derived immature and 22 good quality *in vivo *matured oocytes were collected, COC's were denuded as described above, snap frozen and stored at -80°C until analysis.

### Experiment 1 Microarray analysis of oocyte transcripts

#### a. RNA Extraction, cRNA Preparation, Microarray Hybridization and Processing

5 replicate pools (= days of oocyte collection) each of 100 denuded immature and IVM oocytes were processed for Affymetrix GeneChip analysis. Total RNA was extracted simultaneously from all replicate pools of immature and IVM oocytes using the PicoPure RNA Isolation kit (Arcturus Bioscience, Mountain View, CA, USA), by incorporating a DNase treatment step using RNase-free DNase set (Qiagen, West Sussex, UK), according to the manufacturer's instructions. Quality of the extracted total RNA and concentration (ng/μl) were assessed using the Agilent Bioanalyzer 2100 with RNA 6000 Nano Chip kit (Agilent Technologies, Santa Clara, USA) and Quant-iT™ RiboGreen^® ^RNA assay kit (Invitrogen, Carlsbad, CA), respectively following the manufacturer's instructions. 100 ng of total RNA was subjected to two rounds of linear amplification using the GeneChip^® ^Expression 3'-Amplification Two-Cycle cDNA Synthesis kit (Affymetrix Inc., Santa Clara, CA) according to the manufacturer's instructions. cDNA was synthesized during the first cycle and biotin-labeled nucleotides were incorporated during the second *in vitro *transcription reaction. The resulting labeled antisense RNA samples were fragmented and 15 μg each per array was hybridized to five GeneChip Bovine Genome Arrays (Affymetrix) for 16 h at 45°C. Once completed, arrays were processed according to the manufacturer's protocol and scanned using the GeneChip^® ^Scanner 3000 (Affymetrix).

#### b. Microarray data, Pathway and Gene Ontology Analysis

The Affymetrix GeneChip Bovine Genome array contains 24,027 probe sets corresponding to approximately 23,000 transcripts including assemblies from ~19,000 UniGene clusters. The arrays images were first quantified using Gene Chip Operating Software (GCOS, Affymetrix). The Affymetrix CEL files were loaded into an AffyBatch object using R/Bioconductor [77]. The FARMS algorithm with quantile normalization was used to summarize the probes from the arrays [78]. The non-informative probes were excluded using the informative/non-informative calls from the enhanced-FARMS algorithm [79]. The SAM algorithm [80] with a delta of 0.35 (standard cutoff used by SAM algorithm to determine differentially expressed genes) and a very stringent cut-off with the false discovery rate of 0.0001 were used to identify differentially regulated probe sets between the immature and IVM oocytes. To estimate similarity in gene expression profiles of oocytes at immature and after IVM, samples were subjected to hierarchical clustering. The average linkage clustering algorithm was applied to the logged interpretation of the gene list. The confidence levels were calculated using 100 datasets (bootstrapping). Principal Component Analysis (PCA) algorithm was applied to the gene list using GeneSpring Software (Agilent Technologies). Automatic annotation with standard lists was also performed. The expressed differentially regulated genes were classified according to their gene ontology (GO) [81]: Molecular function, Cellular component and Biological process. In order to understand the relationship between the differentially regulated genes and their functional interaction assessment, enrichment of pathway analysis was carried out using DAVID [82,83] and Ingenuity Pathway Analysis (IPA) software http://www.ingenuity.com.

### Experiment 2 Validation of microarray data using qPCR

Four additional replicate pools, each of 10 immature and IVM oocytes were prepared as described above. The mRNA extraction was performed using the Dynabeads^® ^mRNA DIRECT™ Micro Kit (Invitrogen, Paisley, UK) according to the manufacturer's instructions. Following extraction, cDNA was synthesized in a 40 μl reaction volume using SuperScript^® ^III reverse transcriptase kit (Invitrogen) supplemented with 200 ng of random primers (Invitrogen) according to the manufacturer's instructions. The cDNA synthesis reaction conditions were 70°C for 5 min, 25°C for 5 min, 50°C for 1 h, followed by heat inactivation of the enzyme at 75°C for 15 min.

Relative transcript abundance of selected cell cycle-associated genes was assessed by performing qPCR using the ABI Prism 7300 Sequence Detection System (Applied Biosystems Foster City, CA, USA). Primer sequences and product sizes are described in Table 3. Analysis of qPCR was performed in a 25 μl reaction volume by adding 1.5 μl cDNA (0.30 oocyte equivalent) aliquot of each sample to the PCR mix containing gene specific primers and 50% Power SYBR^® ^Green PCR Master mix (Applied Biosystems). qPCR conditions were 2 min at 50°C, 10 min at 95°C, 40 cycles of 15 s at 95°C and 1 min at 60°C for annealing and extension. At the end of each qPCR reaction, melt curve analysis was performed for all genes to check the specificity of the products. Samples were measured in duplicate for each gene of interest and the reference gene *H2AFZ *[84] was measured in all samples as normalizer.

Quantification of transcript (mRNA expression) levels was carried out by using the comparative quantification cycle (Cq) method (ABI Prism Sequence Detection System, User Bulletin No. 2 (Applied Biosystems) [85]. Normalization was carried out by subtracting the Cq values of *H2AFZ *from the corresponding Cq values of the target gene. Following normalization the relative abundance of mRNAs between the two populations was calculated from the expression ratios of immature and IVM oocytes to calculate a fold change value. Statistical analysis of the expression values was carried out using the student's t-test.

### Experiment 3 Effect of α-amanitin on transcript abundances

The aim of this experiment was to assess the effect of oocyte IVM in the presence of α-amanitin, a transcription inhibitor acting through inhibition of RNA polymerase II [41,57], on transcript abundances. Immature COCs (4 replicates of 200) were collected from slaughterhouse ovaries as described above. On a given day (= replicate) 50 COCs were denuded immediately and snap frozen in pools of 10. The remaining 150 COCs were randomly divided among the following three treatments in groups of 50: (1) matured *in vitro *for 24 h as described above (Control), (2) cultured for 24 h in IVM medium supplemented with 25 μg/ml α-amanitin, and (3) cultured in α-amanitin-free IVM medium for 3 h and then transferred to IVM medium supplemented with 25 μg/ml α-amanitin for the remaining 21 h. Concentration of α-amanitin and length of culture were based on previous publications [42,57]. After maturation, COCs were denuded of their surrounding cumulus cells, snap frozen in pools of 10 per treatment and stored at -80°C until analysis. RNA extraction, cDNA synthesis and qPCR analysis were carried out as described for Experiment 2 above.

### Experiment 4 Effects of analysis procedures on the interpretation of oocyte transcriptional profiles

As mentioned above, there are conflicting reports in the literature [32,36-38] on oocyte transcription profiles during maturation. The aim of these experiments was to examine the effects of certain transcript analysis procedures on the outcomes of the experiment, and if these contributed to the conflicting conclusions. Specifically we examined (1) the type of primers used during cDNA synthesis (Experiment 4a), (2) the expression profiles of some over-expressed genes in *in vitro *matured oocytes after normalization with validated reference genes, (Experiment 4b), and (3) the expression profiles of some over-expressed genes in *in vivo *matured oocytes after normalization with validated reference genes (Experiment 4c). In order to avoid unintentional sources of variations, some modifications were made to the Materials and Methods as described below.

#### a. RNA isolation and reverse transcription

Four replicate (day or collection) pools of 10 oocytes were prepared as described above and processed for each developmental stage (immature and IVM oocytes). During Experiment 4a, 1 pg/oocyte of luciferase mRNA was added, prior to RNA extraction as an exogenous control. Total RNA was isolated using RNeasy Micro kit (Qiagen) with on-column DNase digestion step using an RNase-Free DNase set (Qiagen), according to the manufacturer's instructions.

During Experiment 4a, the eluted total RNA was mixed well and divided into two equal parts for cDNA synthesis, using either random or anchored oligo (dT) primers (Invitrogen). However, for Experiments 4b and 4c, random primers were used during cDNA synthesis. All RNA samples in the same experiment were simultaneously reverse transcribed into cDNA using the SuperScript^® ^III reverse transcriptase kit (Invitrogen), in a final 25-μl reaction volume, and reaction conditions described above in Experiment 2. After cDNA synthesis, 1 μl of cDNA was taken from each sample as template for PCR amplification to check the reverse transcription success and cDNA quality with primers designed to span intron sequences. This procedure was used as a standard during cDNA synthesis for Experiment 4.

#### b. Optimization and qPCR analysis

Subsets of over-expressed genes from the microarray analysis list and reference genes were selected for re-analysis and comparison using qPCR. Bovine sequences for these genes were retrieved from the NCBI Database http://www.ncbi.nlm.nih.gov/ and used to re-design primers that can amplify longer product sizes compared to primers in Experiment 2 and 3, using Primer 3 software http://frodo.wi.mit.edu/primer3/. Intron spanning primer sequences were preferentially selected, and produced as HPSF (High Purity Salt Free) purified primers (MWG Biotech, Ebersberg, Germany). Sources, primer sequences and product sizes are listed in Table 2. Primers were optimized and specificity of amplicons was confirmed by melt curve analysis and fragment sizes were confirmed by agarose gel electrophoresis.

Detection and quantification of the transcripts was assessed following qPCR procedures. Each qPCR reaction consisted of 1.5 μl cDNA template (equivalent to 0.30 oocyte), 0.1-0.3 μM of each primer and 50% Power SYBR^® ^GREEN PCR Master mix (Applied Biosystems) in a final 15-μl reaction volume. Forty five cycles of qPCR was carried out employing the reaction conditions described above. Data were normalized either to the quantity of luciferase (Experiment 4a) or to the geometric averages of three endogenous reference genes (*PPIA, SDHA, YWHAZ*) that were validated in our laboratory (Experiments 4b and 4c). Quantification was carried out using the relative standard curve method (User Bulletin #2, ABI Prism 7700 Sequence Detection System) and the results were reported as relative expression levels (fold change) compared to the calibrator (immature oocyte).

## Authors' contributions

SM, TF, PL and AE designed the experiment. SM and FC performed the experimental work. SM was the primary author of the manuscript with significant contribution from TF and PL. FC, SM, CL and AA collected *in vitro *oocyte samples for analysis. PM and JPM performed the microarray data analysis. All authors read and approved the manuscript.

## Supplementary Material

Additional file 1 **Figure S1: Hierarchical clustering analysis of the less stringent gene list**. Gene Spring software was used to analyze similarities among the 10 replicate samples (vertical bars) across the two treatments. Colours correspond to the level of expression of the detected genes each of which is represented by one horizontal bar. Click here for file

Additional file 2 **Figure S2: Data quality control analysis of GV (yellow) and MII (red) array results showing how the expression data of the ten replicates group together based on maturational status using (A) PCA plots and (B) hierarchically clustering**. Click here for file

Additional file 3 **Table S1: Transcripts over-expressed in IVM oocytes compared to immature oocytes (GV)**. Click here for file

Additional file 4 **Table S2: Transcripts under-expressed in IVM oocytes compared to immature oocytes (GV)**. Click here for file

Additional file 5 **Figure S3: Comparative gene expression profiles of immature and in vitro mature oocytes after total RNA was reverse transcribed with different primers**. The total RNA from these samples was reverse transcribed either with (A) random primer or (B) anchored oligo (dT) primers. In all cases, expression at the immature (GV) stage was taken as calibrator and relative expression levels were described as fold change. Click here for file
