# Supplementary material for: Sequential analysis of global gene expression profiles in immature and in vitro matured bovine oocytes: potential molecular markers of oocyte maturation
Source: BMC Genomics. 2011 Mar 16;12:151. doi: 10.1186/1471-2164-12-151 (PMC3068982; doi:10.1186/1471-2164-12-151)
Supplement: Additional file 2 — Figure S2: Data quality control analysis of GV (yellow) and MII (red) array results showing how the expression data of the ten replicates group together based on maturational status using (A) PCA plots and (B) hierarchically clustering. [file 1471-2164-12-151-S2.DOC]

A .

B.
