# Supplementary material for: Sequential analysis of global gene expression profiles in immature and in vitro matured bovine oocytes: potential molecular markers of oocyte maturation
Source: BMC Genomics. 2011 Mar 16;12:151. doi: 10.1186/1471-2164-12-151 (PMC3068982; doi:10.1186/1471-2164-12-151)
Supplement: Supplementary file 2 [file 1471-2164-12-151-S1.DOC]

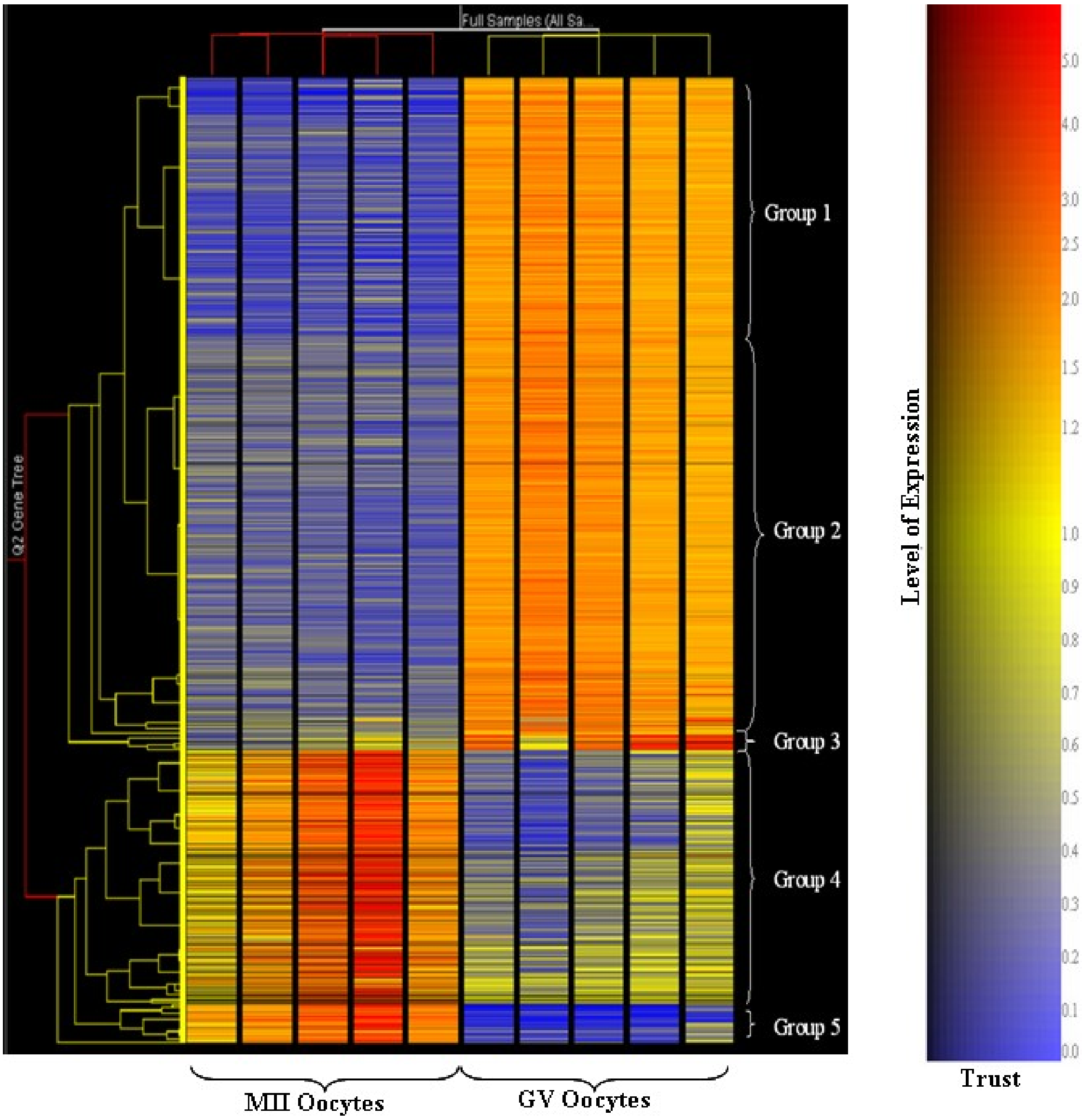

Full Samples (All Sa...
Q2 Gene Tree
Group 1
Group 2
Group 3
Group 4
Group 5
MII Oocytes
GV Oocytes
Level of Expression
5.0
4.0
3.0
2.5
2.0
1.5
1.2
1.0
0.9
0.8
0.7
0.6
0.5
0.4
0.3
0.2
0.1
0.0
Trust
