# Supplementary material for: Sequential analysis of global gene expression profiles in immature and in vitro matured bovine oocytes: potential molecular markers of oocyte maturation
Source: BMC Genomics. 2011 Mar 16;12:151. doi: 10.1186/1471-2164-12-151 (PMC3068982; doi:10.1186/1471-2164-12-151)
Supplement: Supplementary file 3 [file 1471-2164-12-151-S5.DOC]

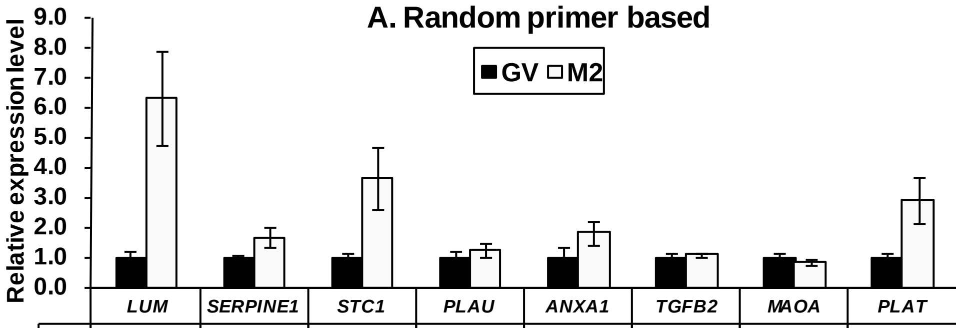

	LUM	SERPINE1	STC1	PLAU	ANXA1	TGFB2	MAOA	PLAT
■ GV	1.0	1.0	1.0	1.0	1.0	1.0	1.0	1.0
□ M2	6.3	1.7	3.6	1.2	1.8	1.1	0.8	2.9

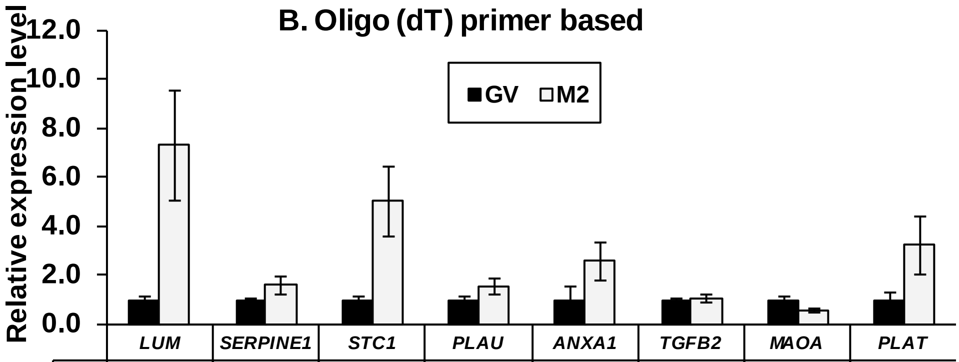

	LUM	SERPINE1	STC1	PLAU	ANXA1	TGFB2	MAOA	PLAT
■ GV	1.0	1.0	1.0	1.0	1.0	1.0	1.0	1.0
□ M2	7.3	1.6	5.0	1.5	2.5	1.1	0.6	3.2
